# Allelic Discrimination of Vitamin D Receptor Polymorphisms and Risk of Type 2 Diabetes Mellitus: A Case-Controlled Study

**DOI:** 10.3390/healthcare11040485

**Published:** 2023-02-07

**Authors:** Amal Ahmed Mohammed, Dina M. Abo El-Matty, Rola Abdel-Azeem, Khaled Raafat, Mona A. Hussein, Amira R. El-Ansary, Wael Hafez, Hatem Ahmed Hassan, Nourelhuda Ahmed Nassar, Nora Mahmoud Selim, Doaa Ghaith, Amal A. El Kholy, Soha M. Abd El Salam, Fatme Al Anouti, Alaa S. Wahba

**Affiliations:** 1 Department of Biochemistry and Molecular Biology, National Hepatology and Tropical Medicine Research Institute (NHTMRI), Cairo 11796, Egypt; 2 Department of Biochemistry, Faculty of Pharmacy, Suez Canal University, Ismailia 41522, Egypt; 3 Department of Internal Medicine, Faculty of Medicine, Ain Shams University, Cairo11566, Egypt; 4 Department of Internal Medicine, National Institute of Diabetes and Endocrinology, Cairo 11562, Egypt; 5 Department of Internal Medicine, Faculty of Medicine, Misr University for Science and Technology, Giza 12577, Egypt; 6 Department of Internal Medicine, The National Research Centre, Cairo 12622, Egypt; 7 Department of Internal Medicine and Gastroenterology, Faculty of Medicine, Minia University, Minia 61519, Egypt; 8 Clinical Pathology Department, Elsahel Teaching Hospital, Cairo 11697, Egypt; 9 Department of Clinical and Chemical Pathology Department, Faculty of Medicine, Cairo University, Cairo 12613, Egypt; 10 Department of Clinical Pharmacy, Faculty of Pharmacy, Ain Shams University, Cairo 11566, Egypt; 11 Department of Medical Microbiology and Immunology, Faculty of Medicine, Suez University, Suez 43512, Egypt; 12 Department of Health Sciences, College of Natural and Health Sciences, Zayed University, Abu Dhabi 144534, United Arab Emirates

**Keywords:** vitamin D receptor, polymorphism, allelic discrimination, T2DM

## Abstract

(1) Background: Type 2 diabetes mellitus (T2DM) is one of the rapidly growing healthcare problems, and several vitamin D receptor (VDR) polymorphisms seem to modulate the risk of T2DM. Our research was designed to investigate the allelic discrimination of VDR polymorphisms and T2DM occurrence risk. (2) Methods: This case-control research included 156 patients with T2DM and 145 healthy control subjects. Most of the study population were males 56.6% vs. 62.8% in the case and control groups, respectively. Genotyping for VDR single nucleotide polymorphisms (SNPs), rs228570 (Fok1), rs7975232 (Apa1), and rs1544410 (Bsm1) was compared between both groups. (3) Results: There was a negative link between vitamin D levels and insulin sensitivity. A significant difference was noted in the allelic discrimination of VDR polymorphism rs228570 and rs1544410 between the study groups (*p* < 0.001). No difference was observed in the allelic discrimination of VDR polymorphism rs7975232 between the groups (*p* = 0.063). Moreover, T2DM patients had significantly higher levels of fasting blood sugar (FBS), glycated hemoglobin HbA1c, 2-h post-prandial blood sugar (PP), serum glutamic oxaloacetic transaminase (SGOT), serum glutamic-pyruvic transaminase (SGPT), total cholesterol, and triglycerides (*p* < 0.001), while High-Density Lipoprotein (HDL) Cholesterol (HDL-C) was significantly decreased (*p* = 0.006). (4) Conclusions: VDR polymorphisms had a positive association with T2DM risk among the Egyptian population. Further large-scale research using deep sequencing of samples is strongly urged to investigate different vitamin D gene variants and interactions, as well as the influence of vitamin D on T2DM.

## 1. Introduction

Diabetes mellitus (DM) is a rapidly growing healthcare challenge due to its impact on morbidity, mortality, and healthcare costs [1]. According to the International Diabetes Federation (IDF), there were about 537 million diabetic subjects reported in 2021. This estimation is likely to extend to 783 million in 2045 [2]. 

Chronic Type 2 Diabetes mellitus (T2DM) is a disorder described by decreased insulin section from the pancreas or increased insulin resistance. It results from the interaction between several genetic and environmental factors, such as obesity, sedentary activities, and hyperlipidemia [3]. 

While the strict causes of T2D are ambiguous, subclinical inflammation and the immune system are considered critical factors in T2D development and consequent complications, such as neuropathy, retinopathy, cardiovascular disorders, and nephropathy [4]. T2D is considered a polygenic and multi-factorial disorder that requires more study of genetic factors from a pathophysiological point of view. Furthermore, lifestyle, and environmental agents should be studied. In recent years, one of the major fields of research has been polymorphisms of probable active genes and the critical role of SNPs in the progress of T2D disorder [5].

Previously, several T2DM genome-wide association (GWAS) reports have presented the complicated polygenic nature of T2DM, which showed that the majority of the identified loci were linked with an elevated susceptibility to T2DM by affecting insulin secretion rather than by decreasing insulin action [3]. This shows that genetic susceptibility has a detrimental effect on the risk of acquiring T2DM.

Fat-soluble vitamin D is present in two forms in the body: D3 (animal origin), and D2 (plant origin). It is a steroid pre-hormone that controls insulin secretion directly or indirectly by regulating Ca^++^ levels. Therefore, low vitamin D levels may have a negative impact on insulin sensitivity and can contribute to the development of insulin resistance [6]. 

Vitamin D deficiency is indicated by total 25(OH)3D serum levels [5]. The global causes of vitamin D deficiency in adults are inadequate sunlight and/or diet. Vitamin D deficiency is frequently identified in diabetes mellitus and is linked to its development and severity [7]. In the serum, vitamin D is carried by vitamin D-binding protein (VDBP), a low-molecular weight glycoprotein (58 kDa) that acts as an active predictor of the bioavailability of (25(OH)3D) levels [8]. Vitamin D is activated by the formation of the VDBP/25(OH)D complex which filtrated and reabsorbed through receptor-mediated uptake in proximal renal tubular cells [9].

For these reasons, diet is a critical factor in T2D development. Genetic polymorphism changes VD absorption or VD receptor functions, as long as it affects the etiology of T2D [10]. In addition, VD activates PPAR-δ (transcriptional factor), which modulates the insulin receptor gene and growing muscle and adipose tissues insulin sensitivity [11]. Additionally, VD controls 3% of the human genome through its receptor (VDR) [12].

VDR is one of the nuclear hormone receptors superfamily with a DNA binding domain, and its gene, set on chromosome 12q13.1 and containing 11 exons [13], is present in all tissues and has several pleiotropic functions [14,15]. FokI (rs2228570), TaqI (rs731236), BsmI (rs1544410), and ApaI (rs7975232) are four major single nucleotide polymorphisms (SNPs) of the VDR gene [16]. Three polymorphisms (ApaI, BsmI, and TaqI) are set at the 3′-end of the VDR gene, which causes silent mutation linked to a rise in VDR mRNA stability. However, the FokI SNP is set at the start codon gives a smaller size and a shorter form protein (424 amino acids), which is more active than the longer (427 amino acids) [17].

Recent GWAS studies have mainly focused on 6 VDR polymorphisms that contribute to the development of several disorders, including T2DM. These polymorphisms include Fok1 in axon 2, Bsm1 in intro 8, Taq1 in axon 9, Apa1 in intron 8, Tru91 in intron 8, and the poly (A) mononucleotide repeat in the 3′-untranslated region [15]. Among them, Fok1 is the only variant associated with the production of a potentially shortened receptor protein, which is more active, and is linked to the risk of developing T2DM [18,19]. 

Furthermore, Fok1, Bsm1, Apa1, and Taq1 have all been shown to affect insulin secretion and sensitivity; thus, VDR polymorphisms are suggested to be a key causal factor in T2DM pathogenesis [20,21,22]. 

These findings suggest that VDR polymorphisms may contribute to type 2 diabetes pathogenesis by their effect on the secretory capacity of β cells, vitamin D concentrations, and T2DM risk and its complications, which are considered an area of interest in recent years. Because of the genetic variation among different populations, the study aimed to investigate whether single nucleotide polymorphisms (SNPs) at the rs228570 (Fok1), rs7975232 (Apa1), and rs1544410 (Bsm1) sites of the VDR gene are linked to susceptibility to T2DM in Egyptian populations. Furthermore, it is vital to study additional genetic polymorphisms to detect persons who are more vulnerable to vitamin D insufficiency and type 2 diabetes in Egyptian populations.

The probable causal factor of vitamin D in T2DM pathogenesis remains not fully known. Therefore, we want to detect extra gene polymorphisms that contribute to T2DM treatment and prevention.

## 2. Materials and Methods

### 2.1. Study Population and Design

This prospective case-control study was carried out in the outpatient clinic at the National Institute of Diabetes and Endocrinology, Cairo, Egypt, from June 2021 to February 2022. All aspects of this study followed the ethical standards of the Declaration of Helsinki [23]. 

Sample size calculation: Based on the study of (Elgazzaz et al., 2016) [24]; VDR gene polymorphisms have an association with T1D and may predispose to the risk of T1D affection, and the haplotype AT was more frequent among diabetics (41%), whereas haplotypes At and aT were more frequent in the control group (39% and 29%, respectively). For a desired power of 0.80 and an alpha error of 0.05, a minimum sample size of 130 subjects per group (total of 260 subjects) will be needed. To compensate for the loss to follow-up, the sample will be increased by 15% to 150 subjects in each group, with a total sample size of 300 subjects. The G*Power© software was used for sample size calculation (Institutfür Experimentelle Psychologie, Heinrich Heine Universität, Düsseldorf, Germany) version 3.1.9.2.

The study included a total of 301 participants who were classified into two groups: 145 healthy subjects as a control group (Group I) and 156 patients with T2DM (Group II). Included patients aged over 18 years old, the group I consisted of healthy individuals only. Group II included type-2 diabetic subjects with or without complications. 

The exclusion criteria included patients with any condition that might interfere with vitamin D metabolism, comprising liver diseases; chronic gastrointestinal diseases; history of bone disorders or fractures; gastric and small bowel resection; or those taking vitamin D supplements or other drugs that increase vitamin D metabolism. In addition, patients with other types of diabetes were excluded.

### 2.2. Data Collection

All participants were interviewed in the Clinics, underwent full clinical examination, and the data collected included demographics (age and gender), lifestyle (smoking), and medical and family history of T2DM via face-to-face interviews. T2DM was defined as fasting blood glucose (FBG) ≥ 126 mmol/L or 2-h post-prandial blood glucose (pp) ≥ 200 or glycated hemoglobin (HbA1C) ≥ 6.5% and/or taking anti-diabetic medication [25]. All participants were screened for FBG, HbA1c, and lipid profiles. Vitamin D levels and VDR polymorphisms were also investigated.

### 2.3. Equipment and Biochemical Measurements

Fifteen milliliters of venous blood was collected into three vacutainer tubes. The first tube was a dry tube to measure FBG, post-prandial blood glucose, and the lipid profile by using Olympus AU400 (Automated biochemistry analyzer, Centeral Valley, PA, USA). The second tube was an ethylenediaminetetraacetic acid (EDTA) tube, which was used to extract deoxyribonucleic acid (DNA) and VDR polymorphism genotyping. The last tube was also an EDTA tube to measure HbA1c, using kits supplied by Teco Diagnostics, Centeral Valley, PA, USA. 

### 2.4. Equipment and Biochemical Measurements

The QIA amp^®^ DNA Blood Mini Kit (QIAGEN GmbH, Hilden, Germany) was used to extract the DNA, which was done in concordance with the manufacturer’s instructions.

The extracted DNA concentration was determined using the Nano Drop^®^ (ND-1000) Spectrophotometer (Nano Drop Technologies Inc., Washington, DC, USA). The absorbance ratio of isolated DNA at 260/280 nm was 1.7–1.9. Genotyping for VDR single nucleotide polymorphisms (SNPs), rs228570, rs7975232, and rs1544410 was performed using real-time polymerase chain reaction (RT-PCR) with TaqMan^®^ allelic discrimination assay software (Applied Bio systems Step One TM Real-Time PCR system Thermal Cycling Block, Singapore) and in coherence with the instructions of the manufacturer. Finally, we tested the association between genetic ancestry and risk after accounting for potential confounding effects.

Vitamin D measurement: methodology, and reference range: 5-hydroxy vitamin D3 (25(OH) D3) analysis using conventional Enzyme-Linked Immuno-Sorbent Assay (ELISA); ELISA sandwich kit Cat. -No: (EIA-3153), DRG International Inc., USA. The determination of absorption was measured with an ELISA reader SATAT FAX. The cut-off for serum 25(OH) was 35 ng/dl (normal if 35 nm/dL, and low if less than 35nm/dL) [26,27]. 

### 2.5. Ethical Approval

The study was approved by the Life Science Ethics Committee of the National Institute of Diabetes and Endocrinology (No: IDE00280). Moreover, informed consent was provided to all participants. 

### 2.6. Statistical Analysis of Data

Data analysis was carried out using statistical software (SPSS), version 23 (SPSS Inc. 233 South Wacker Drive, Chicago, IL, USA). Quantitative variables were represented as mean ± SD or median interquartile range. Comparative analysis was done using the Student’s *t*-test or the Mann–Whitney U test. Using the χ2 goodness-of-fit test, all frequencies in cases and controls were by frequencies predicted by the Hardy–Weinberg equilibrium with a *p* value > 0.05. Categorical data association was tested using the χ2 test for independence. The Monte Carlo method is used in the case of computational limits, and Fisher’s exact test is used when sample sizes are small. A *p*-value ≤ 0.05 was considered statistically significant.

## 3. Results

### 3.1. Demographic and Clinical Features of the Study Population

This study included 145 healthy participants as a control group (Group I), of which 82 (56.6%) were males, aged (38: 64) years, with a mean ±SD of 44.5 ± 5.97. Out of these 145 participants, 21 (14.5%) were smokers, and 100 (69%) were obese. The second group of participants (Group II) comprised 156 patients with T2DM. In this group, there were 98 (62.8%) males aged 30 to 65 years (49 ± 9.2), 18 (11.5%) of whom were smokers, and 126 (80.8%) were obese (Table 1). 

### 3.2. Comparison of the Laboratory Findings between Both Groups

Among the patients who had vitamin D levels <34.5 IU (n = 157), 95 (60.5%) were males, 118 (75.2%) were obese, and 20 (12.7%) were smokers (Table 2).

As shown in Table 3, T2DM patients had significantly higher levels of FBS, HbA1c, PP, SGOT, SGPT, total cholesterol, and triglycerides (*p* < 0.001). In contrast, HDL-C was significantly decreased (*p* = 0.006) (Table 3). In addition, all Laboratory Findings of the Studied Population were provided in Supplementary Materials.

### 3.3. Allelic Discrimination Differences between the Study Groups

Our results revealed a significant variation in the allelic discrimination of VDR polymorphism rs228570 and rs1544410 between the study groups (*p* < 0.001). It was observed that the rs228570 mutant type was present among 2 (1.4%) participants in group I, compared to 45 (28.8%) in group II, and the rs1544410 mutant type was not present in any participant, n = 0 (0.0%), in group I, compared to in group II, where 13 (8.3%) participants had the rs1544410 mutant type. In contrast, there was no significant difference in the allelic discrimination of the VDR polymorphism rs7975232 (*p* = 0.063) (Table 4). Furthermore, multivariate analysis of allelic discrimination of rs228570, comparison between study groups based on rs228570, comparison between study groups based on rs1644410, and comparison between study groups based on rs7975232 were provided in Supplementary Materials.

### 3.4. Comparison of the Study Groups Based on the Vitamin D Status

About 157 (52.2%) of patients had vitamin D levels ≤34.5 IU, and 144 (47.8%) had vitamin D levels >34.5 IU (Table 5).

### 3.5. Receiver Operating Characteristic (ROC) Curve of Vitamin D

**Figure 1:**
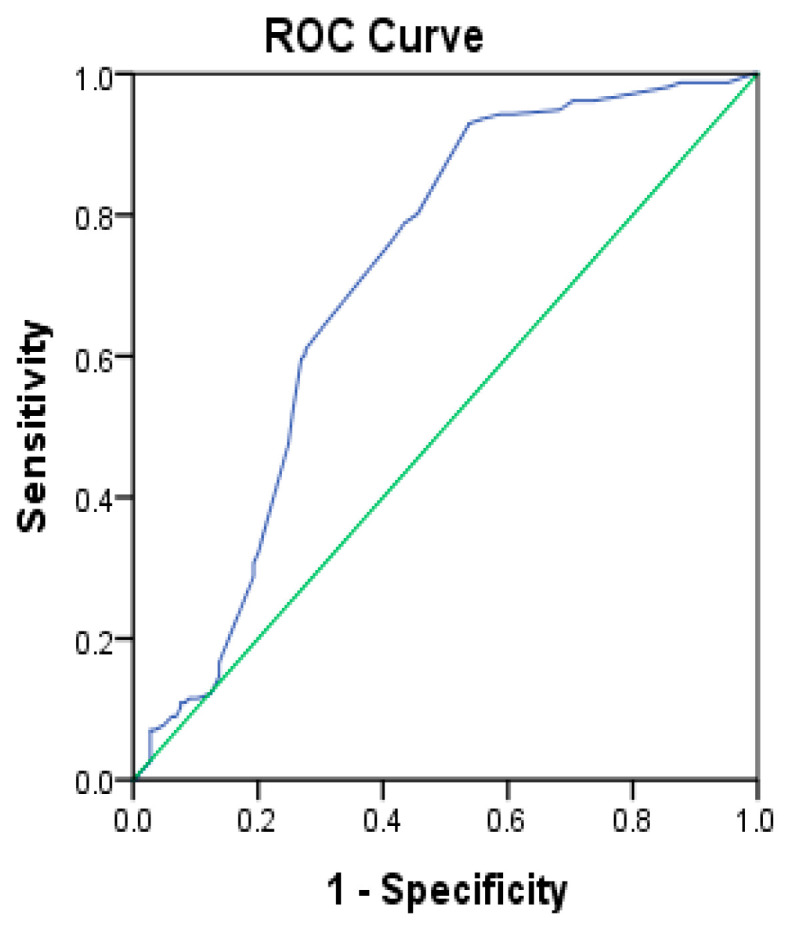
ROC curve of vitamin D.

A receiver operating characteristic, or ROC curve, illustrated that vitamin D had a high significant discriminatory effect in T2DM with AUC = 0.706, *p*-value < 0.001 and 95% CI (0.645-0.767). (Table 6) and (Figure 1).

Additionally, comparison between the study groups based on insulin status, comparison between study groups based on fasting insulin, and roc curve of fasting insulin were provided in Supplementary Materials.

## 4. Discussion

In this case-control study, we aimed to investigate the allelic discrimination of vitamin D polymorphism in T2DM. 

VDR is critically convoluted in endocrine system regulation. As a significance, it is a possible genetic risk factor in metabolic disorder evaluation. In the endocrine system, the VD function is controlled by both genomic and non-genomic approaches [28]. In the genomic setting, ligation of 1,25-dihydroxy vitamin D3 (1,25-(OH)2D3) to the cytosolic/nuclear VDR, which is a member of the steroid/thyroid hormone-receptor superfamily, causes the initiation of other factors that permit the transcription of the equivalent genes [29]. However, a putative membrane VDR triggers the non-genomic pathway, which explains the direct influence of VD [30]. The VDR gene is present on chromosome 12q13.11 and holds 14 exons. In addition, it holds a great promoter area, allowing it to perform numerous transcripts specified to the tissue [31], such as tissues that contribute to glucose metabolism modulation, including pancreatic cells and muscle [32]. Numerous SNPs, such as rs11574129 and rs739837, are present at the 3′-untranslated region (UTR) of the VDR gene; this area is convoluted in gene expression regulation through the adjustment of mRNA steadiness. The FokI polymorphism is present at the 5′-end of the VDR gene and causes T > C disparity at the translation start codon in exon 2 [33]. Furthermore, FokI, BsmI, ApaI, and TaqI affect insulin emission [20] and sensitivity [21]. Therefore, VDR gene polymorphisms are linked to T2D pathogenesis by regulating the pancreatic β-cells’ secretory capacity [22].

The genetic alterations of VDR are critical in T2DM pathogenesis due to Ca metabolism alteration, insulin release, cytokine expression, and adipocyte function [34]. We found a significant difference between the groups in the allelic discrimination of the VDR polymorphisms rs228570 (Fok1) and rs1544410 (Bsm1) but not rs7975232 (Apa1) in the Egyptian population. 

Our results are supported by many previous studies. El Gendy et al. found a positive association in VDR Fok1 polymorphism and no association in Bsm1 polymorphism with T2DM predisposition among the Egyptian population [35]. Moreover, Mackawy et al. reported a link between Fok1 polymorphism and inflammation and insulin resistance in an Egyptian population [36]. Similar findings were also reported in Pakistan, Mexico, China, Kuwait, Turkey, and the United Arab Emirates (UAE) [37,38,39,40,41,42]. Additionally, a recent report described a significant association between Fok1 and Bsm1 but not Apa1 polymorphisms and the risk of T2DM regardless of ethnicity and genotyping techniques [43]. 

In contrast, many studies in the literature disagree with our findings. Erasmus et al. showed no link between Fok1 polymorphism and glycemic status among the South African population [44]. In addition, Bid et al. showed no significant difference in the allelic frequency of VDR polymorphisms, including Fok1, Bsm1, and Taq1, between diabetic patients and healthy subjects in India [45]. Moreover, no associations were reported between T2DM and Fok1 in the Caucasian population, or between Fok1 and Bsm1 in England [46]. The differences between studies could be attributed to the small sample size, small statistical power, heterogeneity in diagnostic approaches, patient characteristics, and ethnic and environmental disparities.

Vitamin D triggers human insulin gene transcription, and its response component is established in the area of the insulin gene promoter, which clarifies its effect on insulin [47]. Additionally, vitamin D insufficiency may be linked to decreased insulin emissions in T2DM. We found a negative linkage between the presence of vitamin D and insulin sensitivity.

Yokoyama et al. suggested similar findings that higher vitamin D levels were linked to the improvement of (CKD) chronic kidney disease outcomes in diabetic patients. This association is regulated by the presence of the Fok1 polymorphism [48]. This suggests that VDR polymorphisms not only contribute to the susceptibility to T2DM, but also the pathogenesis of its complications. In addition, this association between low vitamin D concentrations and T2DM has been supported by many different studies [47,49,50,51].

In addition, our study showed that higher concentrations of vitamin D were significantly linked to lower glucose & insulin concentrations, and Homeostatic Model Assessment for Insulin Resistance (HOMA-IR). In agreement with this finding, Mackawy et al. noted a negative link between insulin and vitamin D concentrations, HbA1c, HOMA-IR, and FBG levels in the Egyptian population [36]. Furthermore, Luo et al. claimed that released inflammatory mediators in diabetic patients were linked to insulin resistance, and the parameters of glycemic control were not returned after taking vitamin D [52]. 

Ma et al. found that the risk of T2DM was associated with the combination of vitamin D lower levels and Fok1 or Taq1 VDR polymorphism existence, disease severity, and coronary artery disease [53]. It was also confirmed that vitamin D deficiency is linked to the reduction of the excretion of insulin from the pancreatic β-cells. This association between decreased vitamin D levels and insulin resistance modulates the immune response, induction of systemic inflammation, and induction of insulin resistance through VDR in the muscles and liver [54,55,56].

In different ethnicities and populations, many studies have tried to reveal the link between T2D susceptibility and VDR gene polymorphisms. However, the results were discrepant from each other due to variances in experimental methods, small sample sizes, little statistical power, clinical heterogeneity, patients’ diagnostic criteria, and interfaces between genetic background and environmental incentives according to geographic variances. A Chinese meta-analysis of 30 case-control studies identified that the BsmI polymorphism was linked to T2D feebly in two genetic models of Bb vs. bb and BB + Bb vs. bb. After analysis of the subgroup, there was a link between the BsmI polymorphism and T2D risk. However, a higher link was found between the FokI polymorphism and T2D in the Chinese population [57].

Another meta-analysis that included 47 publications detected the link between FokI polymorphism and the susceptibility of T2D regarding the dominant model (OR = 1.37), recessive model (OR = 1.10), allelic model (OR = 1.24), ff vs. FF model (OR = 1.36), and Ff vs. FF model (OR = 1.34). Additionally, the BsmI polymorphism heterozygote genotype had a strong link with T2D susceptibility. Nevertheless, TaqI and ApaI were not genetic potentials for T2D [43].

After the subgroup analysis regarding ethnicity, the link of FokI polymorphism to T2D susceptibility was found only in Asians, not in Europeans and Africans. However, in the overall analysis, the TaqI polymorphism was not linked to the risk of T2D. In subgroup analysis, the TaqI polymorphism, in some genetic models, decreases, and in another, increases T2D risks. In the Asian population, the Bb vs. BB genotype of BsmI was linked to the risk of T2D [43].

In an Egyptian study, there was a linkage between vitamin D receptor (VDR) (BsmI) gene polymorphism and genetic T2DM susceptibility. The Bb genotype was higher in the T2DM group (42.5%) than in the control group (12.5%), and the bb genotype was higher in the T2DM group (7.5%) than in the control group (5%), but without statistical significance. The b allele was higher in the T2DM group (28.8%) than in the control group (11.3%). The Bb allele was found to be riskier than the BB allele by 5.610 [confidence interval (CI) = 1.97–15.96], whereas the bb allele was found to be riskier than the BB allele by 2.475 (CI = 0.46–13.09). The b allele was found to be riskier than the B allele by 3.18 (CI = 1.47–6.90) [58].

A recent meta-analysis confirmed the efficacy of vitamin D supplementation on the reduction of insulin resistance when given in large doses for shorter periods, especially in those with vitamin D deficiency and optimal glycemic control at baseline [59]. However, there is a lack of evidence regarding the efficacy or utility of D supplements in the context of T2DM, so it could be recommended to help achieve glycemic control and prevent T2DM complications until otherwise proven. 

Our study noted a critical elevation in cholesterol and triglyceride concentrations and a lowering in HDL-C among diabetic patients compared to non-diabetics. The significant association between T2DM and abnormal lipid profiles is linked to insulin resistance and elevated free fatty acid flux [60]. In contrast, Gendy et al.’s study reported no association between lipid levels and VDR polymorphisms [35]. However, vitamin D is associated with lipid metabolism regulation, fatty acid oxidation, and lipid synthesis inhibition [61].

On the contrary, there are studies describing no relationship between type 2 diabetes mellitus patients and healthy subjects in the allele, as well as genotype frequencies in vitamin D receptor FokI gene polymorphism [62,63,64,65]. Molecular descriptions of the fictional association between the polymorphism of the FokI genotype and T2DM are only partly understood. However, it is difficult to decipher the exact reasons for such discrepancies. A number of possibilities should be measured: genetic trait variations, polymorphism of the VDR gene is separate in a specific population, different ethnicities and geographic areas, T2DM is a multi-factorial disorder and different people could be bare to different geographical factors and genetic susceptibility have caused diverse results. Finally, the unsatisfactory study design may also be the reason, such as limited knowledge of non-random sampling and the prospect of collection bias from the hospital-based case-control study.

There were some limitations to our study. First, the sample size was limited. In addition, there was a lack of investigation of all relevant VDR polymorphisms. Finally, the contribution of vitamin D and VDR polymorphisms to the pathogenesis of T2DM was not examined in this study. Therefore, the link between VDR polymorphisms and the pathogenesis of T2DM remains unclear. To adequately discover any other vitamin D gene variations and interactions related with Type 2 Diabetes Mellitus, deep sequencing of samples would be required. Future large-scale research on this topic is highly recommended.

## 5. Conclusions

In summary, our study showed an inverse association between vitamin D levels and insulin sensitivity. Moreover, there was a significant difference between diabetic and non-diabetic subjects in the allelic discrimination of VDR polymorphisms Fok1 and Bsm1 but not Apa1. Vitamin D polymorphism is crucial in the estimation of the risk of type 2 diabetes and promotes early precautions and prevention strategies; nevertheless, large-scale studies are required to validate these findings and examine the role and appropriate route and dosage of vitamin D supplementation in T2DM patients.

## Figures and Tables

**Table 1:** Clinical Characteristics and Demographics of the Study Population.

	Groups		*p*-Value
	Control (N = 145)	Diabetic (N = 156)	
Age (years)			0.952
≤median value	73 (50.3%)	78 (50.0%)	
>median value	72 (49.7%)	78 (50.0%)	
Gender			0.268
Male	82 (56.6%)	98 (62.8%)	
Female	63 (43.4%)	58 (37.2%)	
Smoking			0.447
No	124 (85.5%)	138 (88.5%)	
Yes	21 (14.5%)	18 (11.5%)	
BMI *			0.018
≤25	45 (31.0%)	30 (19.2%)	
>25	100 (69.0%)	126(80.8%)	

* BMI Body mass index.

**Table 2:** Correlation between Vitamin D Levels and the Clinical characteristics and Demographics of Study Population.

	Vitamin D		*p*-Value
	≤34.5 (n = 157)	>34.5 (n = 144)	
Gender			0.793
Male	95 (60.5%)	85 (59.0%)	
Female	62 (39.5%)	59 (41.0%)	
Age (years)	79 (50.3%)	72 (50.0%)	0.956
≤median value			
>median value	78 (49.7%)	72 (50.0%)	
BMI *	39 (24.8%)	36 (25.0%)	0.975
≤25			
>25	118 (75.2%)	108 (75.0%)	

* BMI Body mass index.

**Table 3:** Laboratory Finding of Studied Groups.

	Groups		*p*-Value
	Control Group (N = 145)	Diabetic Group (N = 156)	
Hb A1C	145 (100.0%)	36 (23.1%)	<0.001
Normal			
Diabetic	0	120 (76.9)%	
FBS	89 (61.4%)	0 (0.0%)	<0.001
Normal			
abnormal	56 (38.6%)	156 (100.0%)	
PP	130 (89.7%)	3 (1.9%)	<0.001
Normal			
abnormal	15 (10.3%)	153 (98.1%)	
SGPT	138 (95.2%)	128 (82.1%)	<0.001
Normal			
abnormal	7 (4.8%)	28 (17.9%)	
SGOT	145 (100.0%)	114 (73.1%)	<0.001
Normal			
abnormal	0 (0.0%)	42 (26.9%)	
LDH (U/L)	112 (77.2%)	120 (76.9%)	0.948
Normal			
abnormal	33 (22.8%)	36 (23.1%)	
ESR (1hr)	32 (22.1%)	31 (19.9%)	0.640
Normal			
abnormal	113 (77.9%)	125 (80.1%)	
INR	32 (22.1%)	31 (19.9%)	0.640
Normal			
abnormal	113 (77.9%)	125 (80.1%)	
Urea	143 (98.6%)	145 (92.9%)	0.016
Normal			
abnormal	2 (1.4%)	11 (7.1%)	
Cholesterol	143 (98.6%)	133 (85.3%)	<0.001
Normal			
abnormal	2 (1.4%)	23 (14.7%)	
TG	125 (86.2%)	39 (25.0%)	<0.001
Normal			
Abnormal	20 (13.8%)	117 (75.0%)	
HDL	2517.2%	117.1%	0.006
Normal			
abnormal	120 (82.8%)	145 (92.9%)	
LDH (U/L)	33 (22.8%)	25 (16.0%)	0.139
Normal			
abnormal	112 (77.2%)	131 (84.0%)	

Hb A1C: Glycated Hemoglobin, FBS: Fasting Blood Sugar, PP: 2-Hr Post-Prandial Blood Sugar, SGPT: serum Glutamate-Pyruvate Transaminase, SGOT: Serum Glutamic Oxaloacetic Transaminase, LDH: Lactate Dehydrogenase, ESR: Erythrocyte Sedimentation Rate, INR: International Normalized Ratio, TG: Triglycerides, HDL: High-Density Lipoprotein, LDL: Low-Density Lipoprotein.

**Table 4:** Allelic Discrimination of Vitamin D among the Study Groups.

	Control Group (N = 145)	Diabetic Group (N = 156)	*p*-Value
rs228570	2 (1.4%)	45(28.8%)	<0.001
mutant type			
wild type	143(98.6%)	111(71.2%)	
rs1544410	145(100.0%)	143(91.7%)	<0.001
mutant type			
wild type	0 (0.0%)	13 (8.3%)	
rs7975232	143(98.6%)	147(94.2%)	0.063
mutant type			
wild type	2 (1.4%)	9 (5.8%)	

**Table 5:** Vitamin D Status among the Study Groups.

Vitamin D Level	Frequency	Percent
≤34.5	157	52.2
>34.5	144	47.8
Total	301	100

**Table 6:** Test Result Variable(s): vitamin D.

Cut-Off Value	Sensitivity	Specificity	Area	Standard Error	*p*-Value	95% Confidence Interval	
						Lower Bound	Upper Bound
34.5	64.1	69.7	0.706	0.031	<0.001	0.645	0.767

## Data Availability

Data are available upon request from the first and corresponding author.

## Supplementary Materials

The following supporting information can be downloaded at: https://www.mdpi.com/article/10.3390/healthcare11040485/s1, Table S1: All Laboratory Findings of Studied Population; Table S2: Multivariate Analysis of Allelic Discrimination of rs228570; Table S3: Comparison between Study Groups based on rs228570; Table S4: Comparison between Study Groups based on rs1644410; Table S5: Comparison between Study Groups based on rs7975232; Table S6: Comparison between Study Groups based on Vitamin D Status; Table S7: Insulin Status Among the Study Groups; Table S8: Comparison between Study Groups based on Fasting Insulin; Figure S1: ROC curve of Fasting Insulin.
