# Supplementary material for: Allelic Discrimination of Vitamin D Receptor Polymorphisms and Risk of Type 2 Diabetes Mellitus: A Case-Controlled Study
Source: Healthcare (Basel). 2023 Feb 7;11(4):485. doi: 10.3390/healthcare11040485 (PMC9956945; doi:10.3390/healthcare11040485)
Supplement: Supplementary file 1 [file healthcare-11-00485-s001.zip › Supplementatry.pdf]

**Supplementary Table S1:** All Laboratory Findings of Studied Population

| | Group | | p-value |
|---|---|---|---|
| | Control (N=145) | diabetic gp (N=156) | |
| Age (years) ≤ median value | 73 (50.3%) | 78 (50%) | 0.952 |
| > median value | 72 (49.7%) | 78 (50%) | |
| Gender male | 82 (56.6%) | 98 (62.8%) | 0.268 |
| female | 63 | 58 | |
| Smoking no | 124 (85.5%) | 138 (88.5%) | 0.447 |
| yes | 21 (14.5%) | 18 (11.5%) | |
| BMI <=25 | 45 (31%) | 30(19.2%) | 0.018 |
| >25 | 100 (69%) | 126 (80.8%) | |
| HB (g/dl) normal | 51 (35.2%) | 60 (38.5%) | 0.555 |
| abnormal | 94 (64.8%) | 96 (61.5%) | |
| RBCs (Million/cm) normal | 54 (37.2%) | 60 (38.5%) | 0.827 |
| abnormal | 91 (62.8%) | 96 (61.5%) | |
| WBCs % normal | 82 (56.6%) | 47 (30.1%) | <0.001 |
| abnormal | 63 (43.4%) | 109 (69.9%) | |
| PLT (thousand/cm) normal | 145 (100%) | 0 (0%) | <0.001 |
| abnormal | 0 (0%) | 156 (100%) | |
| MCH (pg) normal | 104 (71.7%) | 128 (82.1%) | 0.033 |
| abnormal | 41 (28.3%) | 28 (17.9%) | |
| MCHC (g/dl) normal | 42 (56.6%) | 87 (30.1%) | <0.001 |
| abnormal | 63 (43.4%) | 109 (69.9%) | |
| PLT (thousand/cm) normal | 145 (100%) | 0 (0%) | <0.001 |
| abnormal | 0 (0%) | 156 (100%) | |
| MCH (pg) normal | 104 (71.7%) | 128 (82.1%) | 0.033 |
| abnormal | 41(28.3%) | 28 (17.9%) | |
| MCHC (g/dl) normal | 42 (29%) | 87 (55.8%) | |
| abnormal | 103 (71%) | 69 (44.2%) | <0.001 |

| | | | |
|---|---|---|---|
| Monocyte (g/L) normal | 0 (0%) | 4 (2.6%) | |
| abnormal | 145 (100%) | 152 (97.4%) | * |
| Hb A1C normal | 145 (100%) | 36 (23.1%) | <0.001 |
| prediabetic | 0 (0%) | 19 (12.2%) | |
| diabetic | 0 (0%) | 101 (64.7%) | |
| FBS normal | 89 (61.4%) | 0 (0%) | <0.001 |
| abnormal | 56 (38.6%) | 156 (100%) | |
| PP normal | 130 (89.7%) | 3 (1.9%) | <0.001 |
| abnormal | 15 (10.3%) | 153 (98.1%) | |
| GPT normal | 138 (95.2%) | 128 (82.1%) | <0.001 |
| abnormal | 7 (4.8%) | 28 (17.9%) | |
| GOT normal | 145 (100%) | 114 (73.1%) | <0.001 |
| abnormal | 0 (0%) | 42 (26.9%) | |
| LDH (U/L) normal | 112 (77.2%) | 120 (76.9%) | 0.948 |
| abnormal | 33 (22.8%) | 36 (23.1%) | |
| ESR (1hr) normal | 32 (22.1%) | 31 (19.9%) | 0.640 |
| abnormal | 113 (77.9%) | 125 (80.1%) | |
| INR normal | 32 (22.1%) | 31 (19.9%) | 0.640 |
| abnormal | 113 (77.9%) | 125 (80.1%) | |
| Urea normal | 143 (98.6%) | 145 (92.9%) | 0.016 |
| abnormal | 2 (1.4%) | 11 (7.1%) | |
| Cholesterol normal | 143 (98.6%) | 133 (85.3%) | <0.001 |
| abnormal | 2 (1.4%) | 23 (14.7%) | |
| TG normal | 125 (86.2%) | 39 (25%) | <0.001 |
| abnormal | 20 (13.8%) | 117 (75%) | |
| HDL normal | 25 (17.2%) | 11 (7.1%) | 0.006 |
| abnormal | 120 (82.8%) | 145 (92.9%) | |
| LDH (U/L) normal | 33 (22.8%) | 25 (16%) | 0.139 |
| abnormal | 112 (77.2%) | 131 (84%) | |
| rs228570 mutant type | 2 (1.4%) | 45 (28.8%) | <0.001 |
| wild type | 143 (98.6%) | 111 (71.2%) | |

| | | | |
|---|---|---|---|
| rs1644410 wild type | 145 (100%) | 143 (91.7%) | <0.001 |
| mutant type | 0 (0%) | 13 (8.3%) | |
| rs7975232 wild type | 143 (98.6%) | 147 (94.2%) | 0.063 |
| mutant type | 2 (1.4%) | 9 (5.8%) | |

**Supplementary Table S2:** Multivariate Analysis of Allelic Discrimination of rs228570

| | Beta coefficients | Standard error | odds ratio | 95% C.I. for odds ratio | | p-value |
|---|---|---|---|---|---|---|
| | | | | Lower | Upper | |
| BMI | 0.673 | 0.304 | 1.961 | 1.080 | 3.561 | .027 |
| Cholesterol | 2.191 | 0.776 | 8.948 | 1.957 | 40.918 | .005 |
| rs228570 | 3.266 | 0.741 | 26.203 | 6.130 | 112.007 | <0.001 |

**Supplementary Table S3:** Comparison between Study Groups based on rs228570

| | rs228570 | | p-value |
|---|---|---|---|
| | mutant type (n=47) | wild type (n=254) | |
| Age (years) <=median value | 26 | 125 | 0.442 |
| | 55.3% | 49.2% | |
| > median value | 21 | 129 | |
| | 44.7% | 50.8% | |
| Gender male | 31 | 149 | 0.349 |
| | 66.0% | 58.7% | |
| female | 16 | 105 | |
| | 34.0% | 41.3% | |
| Smoking no | 41 | 221 | 0.966 |
| | 87.2% | 87.0% | |
| yes | 6 | 33 | |
| | 12.8% | 13.0% | |
| BMI <=25 | 10 | 65 | 0.530 |

| | | | |
|---|---|---|---|
| | 21.3% | 25.6% | |
| >25 | 37 | 189 | |
| | 78.7% | 74.4% | |
| HB (g/dl)<br>normal | 20 | 91 | 0.380 |
| | 42.6% | 35.8% | |
| abnormal | 27 | 163 | |
| | 57.4% | 64.2% | |
| RBCs (Million/cm)<br>normal | 16 | 98 | 0.556 |
| | 34.0% | 38.6% | |
| abnormal | 31 | 156 | |
| | 66.0% | 61.4% | |
| WBCs %<br>normal | 10 | 119 | 0.001 |
| | 21.3% | 46.9% | |
| abnormal | 37 | 135 | |
| | 78.7% | 53.1% | |
| PLT (thousand/cm)<br>normal | 2 | 143 | <0.001 |
| | 4.3% | 56.3% | |
| abnormal | 45 | 111 | |
| | 95.7% | 43.7% | |
| MCH (pg)<br>normal | 37 | 195 | 0.770 |
| | 78.7% | 76.8% | |
| abnormal | 10 | 59 | |
| | 21.3% | 23.2% | |
| MCHC (g/dl)<br>normal | 26 | 103 | 0.060 |
| | 55.3% | 40.6% | |
| abnormal | 21 | 151 | |
| | 44.7% | 59.4% | |
| Monocyte (g/L)<br>normal | 2 | 2 | * |
| | 4.3% | .8% | |

| | | | |
|---|---|---|---|
| abnormal | 45 | 252 | |
| | 95.7% | 99.2% | |
| CRP (mg/L) normal | 1 | 103 | 0.492 |
| | 50.0% | 72.0% | |
| abnormal | 1 | 40 | |
| | 50.0% | 28.0% | |
| Hb A1C normal | 10 | 171 | <0.001 |
| | 21.3% | 67.3% | |
| prediabetic | 6 | 13 | |
| | 12.8% | 5.1% | |
| diabetic | 31 | 70 | |
| | 66.0% | 27.6% | |
| FBS normal | 1 | 88 | <0.001 |
| | 2.1% | 34.6% | |
| abnormal | 46 | 166 | |
| | 97.9% | 65.4% | |
| PP normal | 1 | 132 | <0.001 |
| | 2.1% | 52.0% | |
| abnormal | 46 | 122 | |
| | 97.9% | 48.0% | |
| GPT normal | 42 | 224 | 0.818 |
| | 89.4% | 88.2% | |
| abnormal | 5 | 30 | |
| | 10.6% | 11.8% | |
| GOT normal | 39 | 220 | 0.509 |
| | 83.0% | 86.6% | |
| abnormal | 8 | 34 | |
| | 17.0% | 13.4% | |
| LDH (U/L) normal | 37 | 195 | 0.770 |
| | 78.7% | 76.8% | |
| abnormal | 10 | 59 | |

| | | | |
|---|---|---|---|
| | 21.3% | 23.2% | |
| ESR (1hr) normal | 13 | 50 | 0.217 |
| | 27.7% | 19.7% | |
| abnormal | 34 | 204 | |
| | 72.3% | 80.3% | |
| INR normal | 13 | 50 | 0.217 |
| | 27.7% | 19.7% | |
| abnormal | 34 | 204 | |
| | 72.3% | 80.3% | |
| Urea normal | 43 | 245 | 0.124 |
| | 91.5% | 96.5% | |
| abnormal | 4 | 9 | |
| | 8.5% | 3.5% | |
| Cholesterol normal | 36 | 240 | <0.001 |
| | 76.6% | 94.5% | |
| abnormal | 11 | 14 | |
| | 23.4% | 5.5% | |
| TG normal | 5 | 159 | <0.001 |
| | 10.6% | 62.6% | |
| abnormal | 42 | 95 | |
| | 89.4% | 37.4% | |
| HDL normal | 7 | 29 | 0.500 |
| | 14.9% | 11.4% | |
| abnormal | 40 | 225 | |
| | 85.1% | 88.6% | |
| LDH (U/L) normal | 9 | 49 | 0.982 |
| | 19.1% | 19.3% | |
| abnormal | 38 | 205 | |
| | 80.9% | 80.7% | |
| rs1644410 wild type | 42 | 246 | 0.020 |
| | 89.4% | 96.9% | |

| | | | |
|---|---|---|---|
| mutant type | 5 | 8 | |
| | 10.6% | 3.1% | |
| rs7975232 wild type | 44 | 246 | 0.278 |
| | 93.6% | 96.9% | |
| mutant type | 3 | 8 | |
| | 6.4% | 3.1% | |
| Control Group | 2 | 143 | <0.001 |
| | 4.3% | 56.3% | |
| Diabetic group | 45 | 111 | |
| | 95.7% | 43.7% | |

## Multivariate (logistic regression model)

| | Beta coefficients | Standard error | odds ratio | 95% C.I. for odds ratio | | p-value |
|---|---|---|---|---|---|---|
| | | | | Lower | Upper | |
| Cholesterol | 1.656 | .441 | 5.238 | 2.208 | 12.427 | <0.001 |

**Supplementary Table S4:** Comparison between Study Groups based on rs1644410

| | rs1644410 | | p-value |
|---|---|---|---|
| | wild type | mutant type | |
| Age (years) <=median value | 146 | 5 | 0.388 |
| | 50.7% | 38.5% | |
| > median value | 142 | 8 | |
| | 49.3% | 61.5% | |
| Gender male | 174 | 6 | 0.305 |
| | 60.4% | 46.2% | |
| female | 114 | 7 | |
| | 39.6% | 53.8% | |
| Smoking No | 251 | 11 | 0.790 |
| | 87.2% | 84.6% | |
| Yes | 37 | 2 | |
| | 12.8% | 15.4% | |

| | | | |
|---|---|---|---|
| BMI <=25 | 70 | 5 | 0.248 |
| | 24.3% | 38.5% | |
| >25 | 218 | 8 | |
| | 75.7% | 61.5% | |
| HB (g/dl) normal | 104 | 7 | 0.195 |
| | 36.1% | 53.8% | |
| abnormal | 184 | 6 | |
| | 63.9% | 46.2% | |
| RBCs (Million/cm) normal | 107 | 7 | 0.225 |
| | 37.2% | 53.8% | |
| abnormal | 181 | 6 | |
| | 62.8% | 46.2% | |
| WBCs % normal | 4 | 0 | * |
| | 1.4% | 0.0% | |
| abnormal | 284 | 13 | |
| | 98.6% | 100.0% | |
| WBCs % normal | 125 | 4 | 0.368 |
| | 43.4% | 30.8% | |
| abnormal | 163 | 9 | |
| | 56.6% | 69.2% | |
| PLT (thousand/cm) normal | 145 | 0 | <0.001 |
| | 50.3% | 0.0% | |
| abnormal | 143 | 13 | |
| | 49.7% | 100.0% | |
| MCH (pg) normal | 224 | 8 | 0.173 |
| | 77.8% | 61.5% | |
| abnormal | 64 | 5 | |
| | 22.2% | 38.5% | |
| MCHC (g/dl) normal | 121 | 8 | 0.164 |
| | 42.0% | 61.5% | |
| abnormal | 167 | 5 | |
| | 58.0% | 38.5% | |
| Monocyte (g/L) normal | 4 | 0 | * |
| | 1.4% | 0.0% | |
| abnormal | 284 | 13 | |
| | 98.6% | 100.0% | |

| | | | |
|---|---|---|---|
| Hb A1C normal | 177 | 4 | 0.068 |
| | 61.5% | 30.8% | |
| prediabetic | 17 | 2 | |
| | 5.9% | 15.4% | |
| diabetic | 94 | 7 | |
| | 32.6% | 53.8% | |
| FBS normal | 89 | 0 | 0.017 |
| | 30.9% | 0.0% | |
| abnormal | 199 | 13 | |
| | 69.1% | 100.0% | |
| PP normal | 133 | 0 | 0.001 |
| | 46.2% | 0.0% | |
| abnormal | 155 | 13 | |
| | 53.8% | 100.0% | |
| GPT normal | 253 | 13 | 0.181 |
| | 87.8% | 100.0% | |
| abnormal | 35 | 0 | |
| | 12.2% | 0.0% | |
| GOT normal | 249 | 10 | 0.332 |
| | 86.5% | 76.9% | |
| abnormal | 39 | 3 | |
| | 13.5% | 23.1% | |
| LDH (U/L) normal | 223 | 9 | 0.491 |
| | 77.4% | 69.2% | |
| abnormal | 65 | 4 | |
| | 22.6% | 30.8% | |
| ESR (1hr) normal | 62 | 1 | 0.230 |
| | 21.5% | 7.7% | |
| abnormal | 226 | 12 | |
| | 78.5% | 92.3% | |
| INR normal | 62 | 1 | 0.230 |
| | 21.5% | 7.7% | |
| abnormal | 226 | 12 | |
| | 78.5% | 92.3% | |
| Urea normal | 275 | 13 | 0.434 |

| | | | |
|---|---|---|---|
| | 95.5% | 100.0% | |
| abnormal | 13 | 0 | |
| | 4.5% | 0.0% | |
| Cholesterol normal | 267 | 9 | 0.003 |
| | 92.7% | 69.2% | |
| abnormal | 21 | 4 | |
| | 7.3% | 30.8% | |
| TG normal | 161 | 3 | 0.020 |
| | 55.9% | 23.1% | |
| abnormal | 127 | 10 | |
| | 44.1% | 76.9% | |
| HDL normal | 35 | 1 | 0.628 |
| | 12.2% | 7.7% | |
| abnormal | 253 | 12 | |
| | 87.8% | 92.3% | |
| LDH (U/L) normal | 56 | 2 | 0.717 |
| | 19.4% | 15.4% | |
| abnormal | 232 | 11 | |
| | 80.6% | 84.6% | |
| Control Group | 145 | 0 | <0.001 |
| | 50.3% | 0.0% | |
| Diabetic group | 143 | 13 | |
| | 49.7% | 100.0% | |
| rs228570 mutant type | 42 | 5 | 0.020 |
| | 14.6% | 38.5% | |
| wild type | 246 | 8 | |
| | 85.4% | 61.5% | |
| rs7975232 wild type | 285 | 5 | <0.001 |
| | 99.0% | 38.5% | |
| mutant type | 3 | 8 | |
| | 1.0% | 61.5% | |

| | Beta coefficients | Standard error | odds ratio | 95% C.I. for odds ratio | | p-value |
|---|---|---|---|---|---|---|
| | | | | Lower | Upper | |

| | | | | | | |
|---|---|---|---|---|---|---|
| Cholesterol | 1.732 | .642 | 5.651 | 1.605 | 19.897 | .007 |

**Supplementary Table S5:** Comparison between Study Groups based on rs7975232

| | rs7975232 | | p-value |
|---|---|---|---|
| | wild type (n=290) | mutant type (n=11) | |
| Age (years) <=median value | 146 | 5 | 0.750 |
| | 50.3% | 45.5% | |
| > median value | 144 | 6 | |
| | 49.7% | 54.5% | |
| Gender male | 173 | 7 | 0.792 |
| | 59.7% | 63.6% | |
| female | 117 | 4 | |
| | 40.3% | 36.4% | |
| Smoking no | 253 | 9 | 0.599 |
| | 87.2% | 81.8% | |
| yes | 37 | 2 | |
| | 12.8% | 18.2% | |
| BMI <=25 | 73 | 2 | 0.599 |
| | 25.2% | 18.2% | |
| >25 | 217 | 9 | |
| | 74.8% | 81.8% | |
| HB (g/dl) normal | 106 | 5 | 0.548 |
| | 36.6% | 45.5% | |
| abnormal | 184 | 6 | |
| | 63.4% | 54.5% | |
| RBCs (Million/cm) normal | 107 | 7 | 0.073 |

| | | | |
|---|---|---|---|
| | 36.9% | 63.6% | |
| abnormal | 183 | 4 | |
| | 63.1% | 36.4% | |
| WBCs %<br>normal | 126 | 3 | 0.287 |
| | 43.4% | 27.3% | |
| abnormal | 164 | 8 | |
| | 56.6% | 72.7% | |
| PLT (thousand/cm)<br>normal | 143 | 2 | 0.043 |
| | 49.3% | 18.2% | |
| abnormal | 147 | 9 | |
| | 50.7% | 81.8% | |
| MCH (pg)<br>normal | 224 | 8 | 0.727 |
| | 77.2% | 72.7% | |
| abnormal | 66 | 3 | |
| | 22.8% | 27.3% | |
| MCHC (g/dl)<br>normal | 123 | 6 | 0.425 |
| | 42.4% | 54.5% | |
| abnormal | 167 | 5 | |
| | 57.6% | 45.5% | |
| Monocyte (g/L)<br>normal | 4 | 0 | * |
| | 1.4% | 0.0% | |
| abnormal | 286 | 11 | |
| | 98.6% | 100.0% | |
| CRP (mg/L)<br>normal | 102 | 2 | 0.371 |
| | 71.3% | 100.0% | |
| abnormal | 41 | 0 | |
| | 28.7% | 0.0% | |
| Hb A1C<br>normal | 177 | 4 | 0.087 |

| | | | |
|---|---|---|---|
| | 61.0% | 36.4% | |
| prediabetic | 19 | 0 | |
| | 6.6% | 0.0% | |
| diabetic | 94 | 7 | |
| | 32.4% | 63.6% | |
| FBS normal | 87 | 2 | 0.399 |
| | 30.0% | 18.2% | |
| abnormal | 203 | 9 | |
| | 70.0% | 81.8% | |
| PP normal | 131 | 2 | 0.077 |
| | 45.2% | 18.2% | |
| abnormal | 159 | 9 | |
| | 54.8% | 81.8% | |
| GPT normal | 256 | 10 | 0.789 |
| | 88.3% | 90.9% | |
| abnormal | 34 | 1 | |
| | 11.7% | 9.1% | |
| GOT normal | 249 | 10 | 0.635 |
| | 85.9% | 90.9% | |
| abnormal | 41 | 1 | |
| | 14.1% | 9.1% | |
| LDH (U/L) normal | 227 | 5 | 0.011 |
| | 78.3% | 45.5% | |
| abnormal | 63 | 6 | |
| | 21.7% | 54.5% | |
| ESR (1hr) normal | 61 | 2 | 0.819 |
| | 21.0% | 18.2% | |
| abnormal | 229 | 9 | |
| | 79.0% | 81.8% | |

| | | | |
|---|---|---|---|
| INR<br>normal | 61 | 2 | 0.819 |
| | 21.0% | 18.2% | |
| abnormal | 229 | 9 | |
| | 79.0% | 81.8% | |
| Urea<br>normal | 277 | 11 | 0.473 |
| | 95.5% | 100.0% | |
| abnormal | 13 | 0 | |
| | 4.5% | 0.0% | |
| Cholesterol<br>normal | 268 | 8 | 0.020 |
| | 92.4% | 72.7% | |
| abnormal | 22 | 3 | |
| | 7.6% | 27.3% | |
| TG<br>normal | 161 | 3 | 0.065 |
| | 55.5% | 27.3% | |
| abnormal | 129 | 8 | |
| | 44.5% | 72.7% | |
| HDL<br>normal | 34 | 2 | 0.517 |
| | 11.7% | 18.2% | |
| abnormal | 256 | 9 | |
| | 88.3% | 81.8% | |
| LDH (U/L)<br>normal | 56 | 2 | 0.926 |
| | 19.3% | 18.2% | |
| abnormal | 234 | 9 | |
| | 80.7% | 81.8% | |
| rs1644410<br>wild type | 285 | 3 | <0.001 |
| | 98.3% | 27.3% | |
| mutant type | 5 | 8 | |
| | 1.7% | 72.7% | |
| Control Group | 143 | 2 | 0.043 |

| | | | |
|---|---|---|---|
| | 49.3% | 18.2% | |
| diabetic | 147 | 9 | |
| | 50.7% | 81.8% | |
| rs228570 mutant type | 44 | 3 | 0.278 |
| | 15.2% | 27.3% | |
| wild type | 246 | 8 | |
| | 84.8% | 72.7% | |

| | Beta coefficients | Standard error | odds ratio | 95% C.I. for odds ratio | | p-value |
|---|---|---|---|---|---|---|
| | | | | Lower | Upper | |
| Cholesterol | 1.519 | .712 | 4.568 | 1.131 | 18.456 | .033 |

## Vitamin D

**Supplementary Table S6:** Comparison between Study Groups based on Vitamin D Status

| | Vitamin D | | p-value |
|---|---|---|---|
| | <=34.5 (n=157) | >34.5 (n=144) | |
| Gender male | 95 (60.5%) | 85 (59%) | 0.793 |
| female | 62 (39.5%) | 59 (41%) | |
| Age (years) <=median value | 79 (50.3%) | 72 (50%) | 0.956 |
| > median value | 78 (49.7%) | 72 (50%) | |
| BMI <=25 | 39 (24.8%) | 36 (25%) | 0.975 |
| >25 | 118 (75.2%) | 108 (75%) | |
| HB (g/dl) normal | 59 (37.6%) | 52 (36.1%) | 0.792 |
| abnormal | 98 (62.4%) | 92 (63.9%) | |
| RBCs (Million/cm) normal | 58 (36.9%) | 56 (38.9%) | 0.728 |
| abnormal | 99 (63.1%) | 88 (61.1%) | |
| WBCs % | 3 (1.9%) | 1 (0.7%) | * |

| | | | |
|---|---|---|---|
| normal | | | |
| abnormal | 153 (98.1%) | 143 (99.3) | |
| PLT (thousand/cm) normal | 101 (64.3%) | 44 (30.6%) | <0.001 |
| abnormal | 56 (35.7%) | 100 (69.4%) | |
| MCH (pg) normal | 122 (77.7%) | 110 (76.4%) | 0.786 |
| abnormal | 35 (22.3%) | 34 (23.6%) | |
| MCHC (g/dl) normal | 59 (37.6%) | 70 (48.6%) | 0.053 |
| abnormal | 98 (62.4%) | 74 (51.4%) | |
| CRP (mg/L) normal | 77 (76.2%) | 27 (61.4%) | 0.067 |
| abnormal | 24 (23.8%) | 17 (38.6%) | |
| LDH (U/L) normal | 121 (77.1%) | 111 (77.1%) | 0.998 |
| abnormal | 36 (22.9%) | 33 2.9%) | |
| Smoking no | 137 (87.3%) | 125 (86.8) | 0.906 |
| yes | 20 (12.7%) | 19 (13.2%) | |
| rs228570 mutant type | 21 (13.4%) | 26 (18.1%) | 0.264 |
| wild type | 136 (86.6%) | 118 (81.9%) | |
| rs1644410 wild type | 154 (98.1%) | 134 (93.1%) | 0.032 |
| mutant type | 3 (1.9%) | 10 (6.9%) | |
| rs7975232 wild type | 154 (98.1%) | 136 (94.4%) | 0.092 |
| mutant type | 3 (1.9%) | 8 (5.6%) | |

Multivariate (logistic regression model)

| | Beta coefficients | Standard error | odds ratio | 95% C.I. for odds ratio | | p-value |
|---|---|---|---|---|---|---|
| | | | | Lower | Upper | |
| rs1644410 | 1.343 | 0.669 | 3.831 | 1.033 | 14.209 | 0.045 |

## Fasting insulin

**Supplementary Figure S1:**ROC curve of Fasting Insulin

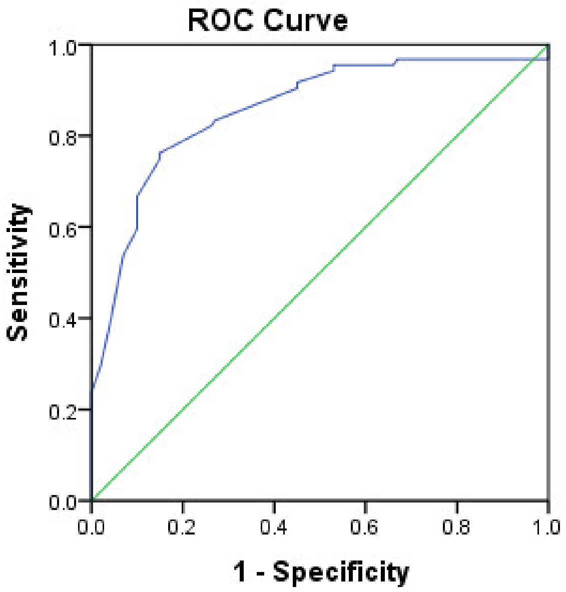


**Area Under the Curve**

Test Result Variable(s): Fasting Insulin

| Area | Standard error | P value | 95% Confidence Interval Lower Bound | 95% Confidence Interval Upper Bound |
|---|---|---|---|---|
| 0.858 | 0.024 | <0.001 | 0.811 | 0.904 |

| Cut off value | Sensitivity | Specificity |
|---|---|---|
| 8.1 | 76.3 | 85 |

**Supplementary Table S7:**Insulin Status Among the Study Groups

| | | Frequency | Valid Percent |
|---|---|---|---|
| Valid | <=8.1 | 122 | 47.7 |
| | >8.1 | 134 | 52.3 |
| | Total | 256 | 100.0 |

**Supplementary Table S8:** Comparison between Study Groups based on Fasting Insulin

| | fasting insulin | | p-value |
|---|---|---|---|
| | <=8.1 | >8.1 | |
| Gender male | 67 (54.9%) | 84 (62.7%) | 0.207 |
| female | 55 (45.1%) | 50 (37.3%) | |
| Age (years) <=median value | 63 (51.6%) | 64 (47.8%) | 0.535 |
| > median value | 59 (48.4%) | 70 (52.2%) | |
| BMI <=25 | 33 (27.0%) | 28 (20.9%) | 0.248 |
| >25 | 89 (73.0%) | 106 (79.1%) | |
| HB (g/dl) normal | 42 (34.4%) | 56 (41.8%) | 0.226 |
| abnormal | 80 (65.6%) | 78 (58.2%) | |
| RBCs (Million/cm) normal | 43 (35.2%) | 57 (42.5%) | 0.232 |
| abnormal | 79 (64.8%) | 77 (57.5%) | |
| WBCs % (n=255) normal | 2 (1.6%) | 0 (0%) | * |
| abnormal | 120 (98.4 %) | 133 (100%) | |
| PLT (thousand/cm) (n=256) normal | 85 (69.7%) | 15 (11.2%) | <0.001 |
| abnormal | 37 (30.3%) | 119 (88.8%) | |
| MCH (pg) (n=256) normal | 94 (77%) | 108 (80.6%) | 0.487 |
| abnormal | 28 (23%) | 26 (19.4%) | |
| MCHC (g/dl) normal | 45 (36.9%) | 72 (53.7%) | 0.007 |
| abnormal | 77 (63.1%) | 62 (46.3%) | |
| CRP (mg/L) (n=100) normal | 58 (68.2%) | 12 (80%) | 0.359 |
| abnormal | 27 (31.8%) | 3 (20%) | |
| LDH (U/L) normal | 91 (74.6%) | 103 (76.9%) | 0.671 |
| abnormal | 31 (25.4%) | 31 (23.1%) | |
| Smoking no | 101 (82.8%) | 116 (86.6%) | 0.401 |

| | | | |
|---|---|---|---|
| yes | 21 (17.2%) | 18 (13.4%) | |
| Vitamin D <=34.5 | 77 (63.1%) | 50 (37.3%) | <0.001 |
| >34.5 | 45 (36.9%) | 84 (62.7%) | |
| rs228570 mutant type | 11 (9%) | 36 (26.9%) | <0.001 |
| wild type | 111 (91%) | 98 (73.1%) | |
| rs1644410 wild type | 118 (96.7%) | 125 (93.3%) | 0.211 |
| mutant type | 4 (3.3%) | 9 (6.7%) | |
| rs7975232 wild type | 120 (98.4%) | 125 (93.3%) | 0.045 |
| mutant type | 2 (1.6%) | 9 (6.7%) | |

Multivariate (logistic regression model)

| | Beta coefficients | Standard error | odds ratio | 95% C.I. for odds ratio | | p-value |
|---|---|---|---|---|---|---|
| | | | | Lower | Upper | |
| rs228570 | 1.329 | 0.382 | 3.779 | 1.786 | 7.993 | 0.001 |
| vit D | 1.069 | 0.267 | 2.913 | 1.727 | 4.913 | <0.001 |
